# Nivolumab as salvage treatment in a patient with HIV-related relapsed/refractory Hodgkin lymphoma and liver failure with encephalopathy

**DOI:** 10.1186/s40425-017-0252-3

**Published:** 2017-06-20

**Authors:** Jose D. Sandoval-Sus, Francis Mogollon-Duffo, Ankita Patel, Nathan Visweshwar, Damian A. Laber, Richard Kim, Michael V. Jagal

**Affiliations:** 1 Division of Hematology Oncology, University of South Florida, Tampa, FL USA; 2 Department of Internal Medicine, University of South Florida, Tampa, FL USA; 3 Section of Satellite Oncology, Moffitt Cancer Center, 12902 USF Magnolia Drive, Tampa, FL 33612 USA; 4 Department of Gastrointestinal Oncology, Moffitt Cancer Center, Tampa, FL USA

**Keywords:** Human immunodeficiency virus, Liver dysfunction, Acute liver failure, Checkpoint inhibitors, Hodgkin lymphoma

## Abstract

**Background:**

We report the first case to our knowledge of a patient with relapsed/refractory classical hodgkin lymphoma and liver failure with encephalopathy along with human immunodeficiency virus/acquired immunodeficiency syndrome infection, successfully treated with nivolumab without major side effects and encouraging prolonged disease control.

**Case presentation:**

In December 2015, at the time of the patient’s progression from his Hodgkin lymphoma after fourth line treatment, he developed persistent fevers, abdominal distension, jaundice and worsening of his liver function tests. Magnetic resonance imaging of abdomen/pelvis demonstrated hepatomegaly with innumerable new liver lesions, splenomegaly with multiple splenic nodules and several new mediastinal, intraperitoneal and retroperitoneal lymphadenopathy. In accordance with the patient’s wishes before admission, and after agreement with the family, nivolumab (3 mg/kg every 2 weeks) was given. Of note, antiretroviral therapy was on hold due to liver function tests, his viral load was undectable and cluster of differentiation 4 counts were 103/uL at the time of nivolumab administration. One week after the first dose of nivolumab both his hepatic encephalopathy and constitutional symptoms started to improve, and after 2 doses, (January 2016) his LFTs were almost back to normal. After 5 months of nivolumab treatment (10 doses), restaging (computerized tomography scans of neck, chest, abdomen, pelvis) done on May 2016 showed resolution of hepatosplenomegaly with two residual small hepatic lesions, heterogeneous spleen with no splenic lesions, and stable non-enlarged retroperitoneal lymph nodes without intraabdominal lymphadenopathy; consistent with partial response.

**Conclusions:**

We report a case of a patient with human immunodeficiency virus/acquired immunodeficiency syndrome -related relapsed/refractory classical Hodgkin lymphoma and acute liver failure with encephalopathy successfully treated with nivolumab after failing all standard therapeutic options. Unlike classic cytotoxic chemotherapy, which relies on preserved organ function to ameliorate potential severe side effects (i.e. myelosuppression), elimination of monoclonal antibodies is fairly independent of baseline renal and hepatic function since they are usually metabolized by circulating phagocytes and/or by their target antigen-expressing cell.

## Background

The interaction between the program cell death-1 (PD-1) located in tumor-specific lymphocytes, and its ligands PD-L1/PD-L2 expressed by neoplastic cells, is one of the main mechanism used by multiple malignancies to induce T-cell immune dysfunction after chronic tumoral immune activation [1, 2]. The development of monoclonal antibodies (mAB) capable of blocking the immunosuppressive signals of these checkpoints, such as the anti-PD-1 mAB nivolumab and pembrolizumab, have demonstrated astonishing clinical activity in a myriad of advance cancers refractory to cytotoxic chemotherapeutic regimens; including hematologic malignancies [3–7]. The malignant Reed-Stenberg (RS) cells found in classical Hodgkin lymphoma (cHL) induce a chronic inflammatory tumoral microenvironment and heavily overexpresses both PD-L1 and PD-L2 [2, 8]. cHL often overexpresses PD-L1 due to 9p24.1 amplification [9]. Phase I/II clinical trials of anti-PD-1 mAB in patients with relapsed/refractory (R/R) cHL have demonstrated the highest overall response rates (ORR) amongst all tumors treated with checkpoint inhibitors (ORR: 65%–87%), with most responses lasting >6 months [6, 10, 11]. Based on these outcomes nivolumab received accelerated FDA approval for the treatment of R/R cHL progressing after autologous stem cell transplantation (ASCT) and brentuximab-vedotin (BV), treatment in May 2016. Nonetheless, all of the three anti-PD-1 pivotal trials in R/R HL excluded patients with human immunodeficiency virus (HIV) infection and/or with acquired immunodeficiency syndrome (AIDS) due to concerns of worsening retroviral control after manipulation of regulatory elements of the immune system. Also, as is the case with the majority of oncologic studies testing novel agents, cHL patients with limited organ function were not included in any of the checkpoint inhibitor trials.

**Fig. 1:**
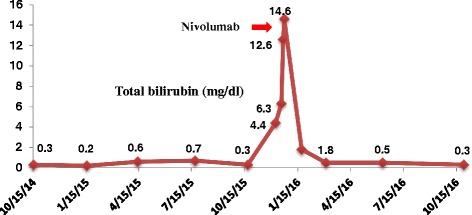
Nivolumab and Bilirubin

**Fig. 2:**
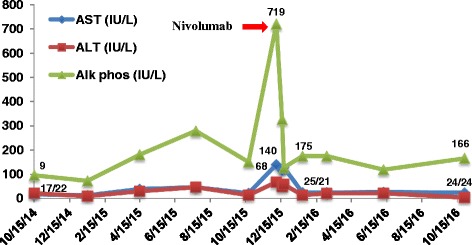
Nivolumab and Liver Function Tests

We report the first case to our knowledge of a patient with R/R cHL and liver failure with encephalopathy along with HIV/AIDS infection, successfully treated with nivolumab without major side effects and encouraging prolonged disease control.

## Case presentation

Forty two-year-old man presented with diaphoresis, unintentional 20-lb weight loss and progressive cervical, axillary, and inguinal lymphadenopathy (LAD) in September 2014. A CT of the neck, thorax, abdomen, and pelvis (NTAP) revealed diffused bilateral cervical, left axillary, retroperitoneal, pelvic, and bilateral inguinal lymphadenopathy (largest LAD: 2.7 cm on left axilla). A left inguinal lymph node excisional biopsy showed replacement of normal lymph node architecture by two distinct Epstein-Barr virus (EBV) positive lymphomas: Burkitt lymphoma (CD20+, CD10+, BCL-2-, MUM-1+) with Ki-67% of 100% and positive t(8;14)(q24;32) in 29% of the cells, and Reed Stemberg cells positive for CD30 and in-situ hybridization for EBV (EBER), consistent with cHL. Bone marrow aspirate and biopsy (BMBx) was infiltrated by cHL (CD15+, CD30+, EBER+, PAX5+, CD20 -). He was diagnosed with HIV on December 2013 and was on antiretroviral therapy (ART) since February 2014. At diagnosis of his lymphomas, his HIV-1 viral load was undetectable and his CD4 count was 155/uL. From October 2014 to February 2015 he received six cycles of dose adjusted etoposide, prednisone, vincristine, cyclophosphamide, doxorubicin, and rituximab (DA-EPOCH-R) with intrathecal methotrexate and cytarabine prophylaxis every cycle. After completion of EPOCH-R, a CT of NTAP showed interval development of multiple liver and splenic lesions, retroperitoneal and left axillary LAD enlargement, and resolution of cervical nodes. Liver function tests (LFTs) were normal. Core needle biopsy of a liver nodule depicted cHL with no evidence of Burkitt lymphoma. BMBx had residual cHL cells (<3%) and no Burkitt lymphoma. He received three cycles of salvage chemotherapy with ifosfamide, carboplatin, and etoposide (ICE) from February 2015 to April 2015. He was hospitalized on April 2015 with persistent fevers and malaise. CT scans confirmed progression of liver lesions with normal alanine transaminase (ALT) (3.0 mg/dl, normal range (NR): normal range: 5.0–55 U/L) and total bilirubin (TB) (0.6 mg/dl, NR: 0.2–1.2), with mild elevation of both alkaline phosphatase (AP) (181 mg/dl, NR: 40–150 U/L) and aspartate aminotransferase (AST) (39 mg/dl, NR: 5.0–34 U/L). Due to evidence of progressive disease (PD) he was switch to single agent Brentuximab-vedotin and received four doses from April 2015 to June 2015 with several delays due to cytopenias, before being readmitted with new evidence of PD on CT NCAP and on BMBx (cHL involving 70–80% on bone marrow without evidence of Burkitt lymphoma). His LFTs began to deteriorate (AST 93 IU, ALT 81 IU/L, AP 436 IU/L, and TB 1.1. mg/dl). Fourth line salvage chemotherapy with gemcitabine and cisplatin (four cycles) was given from August 2015 to October 2015 with initial resolution of constitutional symptoms and LFTs improvement, but complicated by prolonged thrombocytopenia needing frequent platelet transfusions. Restaging images in November 2015 showed PD with multiple new splenic lesions and retroperitoneal lymph node enlargement. BMBx showed persistent cHL (20% of bone marrow) with no other malignancy. In December 2015, at the time of this progression, he developed persistent fevers, abdominal distension, jaundice and worsening of his LFTs (AST 140 IU/L, ALT 68 IU/L, AP 719 IU/L and TB 4.4 mg/dl). magnetic resonance imaging (MRI) of abdomen/pelvis demonstrated hepatomegaly with innumerable new liver lesions, splenomegaly with multiple splenic nodules and several new mediastinal, intraperitoneal and retroperitoneal LAD. The patient developed severe hepatic encephalopathy in concordance with worsening liver function (AST 129 IU/L, ALT 61 IU/L, AP 127 IU/L, albumin 2.2 mg/dl, PTT 54.4 s, PT 33.2 s, INR 2.2 and TB 14.6 mg/dl). The patient’s HIV viral load at that time was undectable with less than 40 copies/ml detected. At this moment the options were either comfort care in hospice or treatment with nivolumab. In accordance with the patient’s wishes before admission, and after agreement with the family, nivolumab (3 mg/kg every 2 weeks) was given. Of note, ART was on hold due to LFTs, and CD4 counts were 103/uL at the time of nivolumab administration. One week after the first dose of nivolumab both his hepatic encephalopathy and constitutional symptoms started to improve, and after 2 doses, (January 2016) his LFTs were almost back to normal (AST 26 IU/L, ALT 15 IU/L, AP 157 IU/L and TB 1.3 mg/dl) (Fig. 1). He was able to restart his ART (CD4 count on 2/2016: 157/uL). Following 3 months of nivolumab treatment, a BMBx on March 2016 showed variable marrow cellularity with low level involvement of cHL (5%). After 5 months of nivolumab treatment (10 doses), restaging CT scan of NCAP done on May 2016 showed resolution of hepatosplenomegaly with two residual small hepatic lesions, heterogeneous spleen with no splenic lesions, and stable non-enlarged retroperitoneal lymph nodes without intraabdominal LAD; consistent with partial response (PR). At the time of his last follow up on December 2016 (29 months after initial diagnosis and 12 months after first nivolumab dose) he continues to be asymptomatic without constitutional symptoms eastern cooperative oncology group (ECOG) performance status of 1, with normal LFTs (AST 26 IU/L, ALT 15 IU/L, AP 157 IU/L and TB 1.3 mg/dl) and only mild thrombocytopenia (126.000/mm^3^) (Fig. 2). Importantly, both HIV-1 viral load and CD4 counts have remain stable on ART (undetectable and 126/uL, respectively), and he has not experience any immune related adverse events (irAEs) after 24 doses of nivolumab on his continued therapy with ART regimen (elvitegravir, cobicistat, emtricitabine and tenofovir.)

## Discussion and conclusions

The ability to manipulate neoplastic signals of immune tolerance with targeted biological compounds, such as mab, has certainly changed the therapeutic paradigm of solid and hematologic malignancies. Unfortunately the landmark clinical trials of checkpoint inhibitors across multiple oncologic disorders have systematically excluded patients with advanced organ failure and/or active viral infections that could derive in unpredictable immune alterations after exposure to these agents [6, 10–15]. We report a case of a patient with HIV/AIDS-related R/R cHL and acute liver failure with encephalopathy successfully treated with nivolumab after failing all standard therapeutic options. Although there have been previous reports of patients with solid tumors treated with anti-PD-1 inhibitors while having limited organ function [16] or with ongoing chronic viral infections (i.e. HCV/HIV) [17], there has not been reported cases of anti-PD-1 treatment in a patient with a R/R hematologic malignancy, while experiencing both grade 4 hepatic dysfunction and advanced HIV with CD4 counts persistently below 200/uL. Unlike classic cytotoxic chemotherapy, which relies on preserved organ function to ameliorate potential severe side effects (i.e. myelosupression), elimination of mab is fairly independent from baseline renal and hepatic function since they are usually metabolized by circulating phagocytes and/or by their target antigen-expressing cell [18]. Although it has not been systematically tested in prospective clinical studies, these distinct pharmacokinetic properties could expand the use of checkpoint antibody inhibitors in oncologic patients with severe organ dysfunction. Checkpoint inhibitors do convey class-specific immune related adverse events (irAEs), such as pneumonitis, hepatitis and colitis, which may be exacerbated by poor baseline renal and/or hepatic reserve. Kanz et al. recently reported a multicenter retrospective analysis of patients with advanced solid tumors and suboptimal cardiac (left ventricular ejection fraction <45%), renal (creatinine >2 mg/dl or GFR < 30 ml/min) and/or hepatic (AST, ALT and/or bilirubin ≥3 × ULN) function; treated with either nivolumab or pembrolizumab [16]. From the 27 patients analyzed, 7 had hepatic dysfunction and 4 had cirrhosis. Importantly, none of these patients had evidence of life-threatening encephalopathy and acute liver failure. Four patients had moderate hyperbilirubinemia (highest level = 4 mg/dl) and AST elevation (highest level = 150 mg/dl). Two patients with baseline kidney injury experienced irAEs (grade 3 hepatitis), which resolved with corticosteroids, and one subject with chronic kidney injury had unexplained recurrent ascites of unclear etiology without mention of worsening liver function. Notably, 48% experienced treatment benefit, and one melanoma patient with cirrhosis, renal dysfunction and ECOG performance status of 3 had a complete response at four months post pembrolizumab therapy [16].

Immune checkpoints play an important role in immunologic homeostasis; hence, patients with chronic conditions such as HIV/AIDS, have been classically precluded from clinical trials involving immunotherapy due to concerns of unforeseen adverse events in the treated host [17]. The PD-1/PDL-1/PD-L2 immune inhibitory pathway has been implicated in the pathogenesis of chronic HIV infection. During chronic HIV infection, virus-specific CD 8^+^ T-cells expressing high levels of PD-1 are characterized by lower cell proliferation and suboptimal cytokine production, functions that may not be appropriately restored by ART [19]. Interestingly, studies in mice and SIV primate models showed immune exhaustion of in virus-specific T cells, which was effectively revamped by the blockade of the PD-1/PDL-1/PDL-2 inhibitory axis [19]. Given these insights from the immune virology realm, our current fears of plausible immune dysregulation in chronic viral immunosuppressant infections may be erroneous. Checkpoint inhibitors could potentially be tested in HIV infected hosts to enhance immune effector responses. [19] This concept is currently undergoing evaluation through a phase I trial using a PDL-1 inhibitor in HIV patients on ART (NCT02028403). Davar D et al. described the only clinical experience of PD-1 inhibitors for the treatment of advance solid tumors in patients with chronic retroviral infections [17]. They reported an HIV+/HCV+ patient with R/R stage IV BRAF V600E mutated melanoma who was treated with pembrolizumab after failing several lines of treatment. Although he had a poor tumoral control with the PD-1 inhibitor, throughout treatment he did not experience any major interactions with his ART therapy with good control of his viremia, and he did not experience any irAEs.

To our knowledge, our case is the first reported patient with hepatic encephalopathy due to an advanced refractory HIV-related hematologic malignancy successfully, and safely, treated with PD-1 inhibitors. Clinical trials to explore the benefits of these immunotherapies in the HIV population with advanced solid tumors should be supported (NCT02408861). Our case suggests that HIV-related Hodgkin lymphoma could be an optimal disease for these agents to be tested on, due to the exquisite activity of anti-PD-1 antibodies in this disease [2, 10].

## Abbreviations

AIDS: Acquired immunodeficiency syndrome
ALT: Alanine transaminase
AP: Alkaline phosphatase
ART: Antiretroviral therapy
ASCT: Autologous stem cell transplantation
AST: Aspartate aminotransferase
BMBx: Bone marrow aspirate and biopsy
BV: Brentuximab-vedotin
CD: Cluster of differentiation
cHL: Classical Hodgkin lymphoma
DA-EPOCH-R: Dose adjusted etoposide, prednisone, vincristine, cyclophosphamide, doxorubicin, and rituximab
EBER: In-situ hybridization for EBV
EBV: Epstein-Barr virus
ECOG: Eastern cooperative oncology group
HCV: Hepatitis C Virus
HIV: Human immunodeficiency virus
ICE: Ifosfamide, carboplatin, and etoposide
irAEs: Immune related adverse events
LAD: Lymphadenopathy
LFTs: Liver function tests
mAB: Monoclonal antibodies
MRI: Magnetic resonance imaging
NR: Normal range
NTAP: Computerized tomography of the neck, thorax, abdomen, and pelvis
ORR: Overall response rates
PD: progressive disease
PD-1: Program cell death-1
PR: Partial response
R/R: Relapsed/refractory
RS: Reed-Stenberg
TB: Total bilirubin
ULN: Upper limit of normal

## References

[1] Nastoupil LJ, Neelapu SS (2015). Novel immunologic approaches in lymphoma: unleashing the brakes on the immune system. Curr Oncol Rep.

[2] Ansell SM (2016). Where Do Programmed Death-1 Inhibitors Fit in the Management of Malignant Lymphoma?. J Oncol Pract.

[3] Topalian SL, Hodi FS, Brahmer JR (2012). Safety, activity, and immune correlates of anti-PD-1 antibody in cancer. N Engl J Med.

[4] Le DT, Uram JN, Wang H (2015). PD-1 Blockade in Tumors with Mismatch-Repair Deficiency. N Engl J Med.

[5] Mahoney KM, Freeman GJ, McDermott DF (2015). The Next Immune-Checkpoint Inhibitors: PD-1/PD-L1 Blockade in Melanoma. Clin Ther.

[6] Ansell SM, Lesokhin AM, Borrello I (2015). PD-1 blockade with nivolumab in relapsed or refractory Hodgkin's lymphoma. N Engl J Med.

[7] Ferris RL, Blumenschein G Jr, Fayette J, et al. Nivolumab for Recurrent Squamous-Cell Carcinoma of the Head and Neck. N Engl J Med. 2016; 10.1056/NEJMoa1602252 PMC5564292 27718784

[8] Armand P (2015). Immune checkpoint blockade in hematologic malignancies. Blood.

[9] Green MR, Monti S, Rodig SJ (2010). Integrative analysis reveals selective 9p24. 1 amplification, increased PD-1 ligand expression, and further induction via JAK2 in nodular sclerosing Hodgkin lymphoma and primary mediastinal large B-cell lymphoma. Blood.

[10] Armand P, Shipp MA, Ribrag V, et al. Programmed Death-1 Blockade With Pembrolizumab in Patients With Classical Hodgkin Lymphoma After Brentuximab Vedotin Failure. J Clin Oncol. 2016; 10.1200/JCO.2016.67.3467 PMC5791838 27354476

[11] Younes A, Santoro A, Shipp M (2016). Nivolumab for classical Hodgkin's lymphoma after failure of both autologous stem-cell transplantation and brentuximab vedotin: a multicentre, multicohort, single-arm phase 2 trial. Lancet Oncol.

[12] Wolchok JD, Kluger H, Callahan MK (2013). Nivolumab plus ipilimumab in advanced melanoma. N Engl J Med.

[13] Borghaei H, Paz-Ares L, Horn L (2015). Nivolumab versus Docetaxel in Advanced Nonsquamous Non-Small-Cell Lung Cancer. N Engl J Med.

[14] Motzer RJ, Escudier B, McDermott DF (2015). Nivolumab versus Everolimus in Advanced Renal-Cell Carcinoma. N Engl J Med.

[15] Reck M, Rodriguez-Abreu D, Robinson AG, et al. Pembrolizumab versus Chemotherapy for PD-L1-Positive Non-Small-Cell Lung Cancer. N Engl J Med. 2016; 10.1056/NEJMoa1606774 27718847

[16] Kanz BA, Pollack MH, Johnpulle R (2016). Safety and efficacy of anti-PD-1 in patients with baseline cardiac, renal, or hepatic dysfunction. J Immunother Cancer.

[17] Davar D, Wilson M, Pruckner C, Kirkwood JM (2015). PD-1 Blockade in Advanced Melanoma in Patients with Hepatitis C and/or HIV. Case Rep Oncol Med.

[18] Mould DR, Sweeney KR (2007). The pharmacokinetics and pharmacodynamics of monoclonal antibodies--mechanistic modeling applied to drug development. Curr Opin Drug Discov Dev.

[19] Velu V, Shetty RD, Larsson M, Shankar EM (2015). Role of PD-1 co-inhibitory pathway in HIV infection and potential therapeutic options. Retrovirology.

